# A Low-Profile and High-isolated MIMO Antenna for 5G Mobile Terminal

**DOI:** 10.3390/mi11040360

**Published:** 2020-03-30

**Authors:** Rongqiang Li, Zixu Mo, Haoran Sun, Xiaofeng Sun, Guohong Du

**Affiliations:** College of Electronic Engineering, Chengdu University of Information Technology, Chengdu 610225, China

**Keywords:** 5G communication, mobile terminal, multiple-input multiple-output (MIMO) antenna, high isolation

## Abstract

An eight-element multiple-input multiple-output (MIMO) frame antenna array in the 3.5 GHz band (3400–3600 MHz) for 5G mobile terminal systems was presented. By using the adjacent grounding and electromagnetic coupling feeding technology, the loop antenna element could generate two resonant frequencies, thus effectively expanding its bandwidth. By adopting double-sided parallel strip line (DSPSL) technology, the electromagnetic coupling inside the loop antenna could be adjusted, and the size of the loop antenna could be effectively reduced so that the MIMO antenna array could obtain a low-profile structure. The total size of the MIMO array was 150 mm × 75 mm × 5.3 mm. Without additional isolation measures, the measured −6 dB impedance bandwidth (BW) was 3400–3660 MHz, and the minimum isolation between antenna elements was better than −20 dB. The proposed antenna was expected to be applied to 5G mobile terminals based on its low-profile, high-isolated characteristics, and good MIMO performance.

## 1. Introduction

As multiple-input multiple-output (MIMO) antenna system can increase channel capacity and transmission rate under the condition of fixed communication bandwidth [1,2,3], various multi-element MIMO antenna arrays have been widely concerned for a mobile communication system. Compared with antennas in a 4G mobile communication terminal system [4,5,6], a MIMO antenna array for a 5G system requires more than six antenna units. They usually work in the microwave frequency band below 6 GHz, such as 3400–3600 MHz, 4800–5100 MHz, and 5700–5800 MHz.

In 5G mobile communication terminal systems, the design of multi-element MIMO antennas is very challenging. Due to the limited space of mobile communication terminals, the main challenge of MIMO antenna design is to arrange multiple antenna elements in a small space, which requires that the antenna elements should have a smaller size and have better isolation between the antenna elements. Some MIMO antennas for 5G smartphone terminals have been proposed. The types of antenna elements selected are mainly inverted F antennas [7,8,9,10], loop antennas [11,12,13,14], and slot antennas [15,16,17,18].

In order to improve the isolation between MIMO antenna elements, the most direct method is to increase the distance between antenna elements. However, due to the limited area of mobile terminal system, some additional decoupling methods are often needed, mainly including neutral line [19,20], parasitic strip [21,22], slotted ground [23,24], the polarized antenna [25,26], self-isolated antenna elements [27,28,29], etc. Zhao et al. [27] proposed an eight-element MIMO array, which consists of a T-shaped feeding element and two symmetrical L-shaped radiating elements, where the two L-shaped elements not only act as the antenna radiating element but also the decoupling element between two adjacent antenna elements. Therefore, the proposed MIMO antenna can achieve very good isolation better than −20 dB. In [28], an eight-element MIMO antenna array with a self-isolated characteristic is proposed for 5G mobile application. The radiating antenna element is an inverted U-shaped radiating structure that is grounded at both ends and includes two vertical open stubs. Isolation better than −19 dB between two antenna elements can be realized for this MIMO antenna. In [29], Ren et al. proposed a dual-band four-element MIMO antenna array, including two self-decoupled antenna pairs. The MIMO antenna has isolation better than −17.5 dB for the low band and −20 dB for the high band. In summary, the above three self-isolated MIMO antennas use grounded elements to improve the isolation of the antenna, and they can achieve isolation better than −17.5 dB. It should be noted that all three antennas are frame antennas, and all frames are higher than 6.2 mm, which is not conducive to the realization of ultra-thin antennas. In addition, some metal frame antennas have also been proposed for 5G terminal communication [30,31].

In this paper, we proposed a low-profile, high-isolated MIMO antenna array with a non-metal frame that operates in the 3400–3600 MHz frequency band. It consisted of eight miniaturized loop antenna elements, which were evenly distributed on two small dielectric substrates perpendicular to the main substrate. Compared with other bezel antennas, the proposed MIMO antenna array had lower sides and similar high isolation characteristics and was suitable for application in ultra-thin mobile phone terminal systems.

## 2. Antenna Design

### 2.1. MIMO Antenna Array Structure

**Figure 1:**
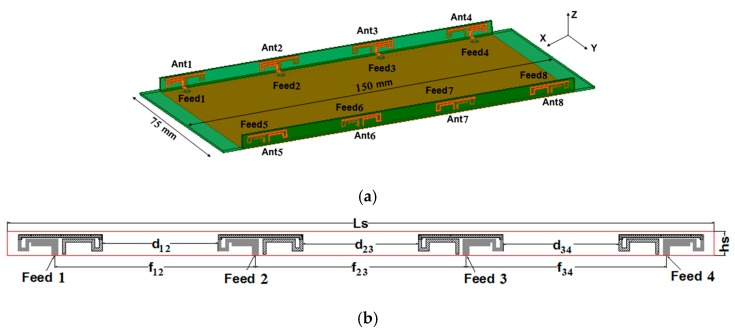
Perspective and side views of the proposed MIMO antenna array (unit: mm). (**a**) Perspective view. (**b**) Side view.

Figure 1a shows the structure of the proposed MIMO antenna array. This array consisted of a system substrate and two small substrates placed vertically on its left and right sides. The dimensions of the system substrate and each small substrate were 150 mm × 75 mm × 0.8 mm and 134 mm × 4.5 mm × 0.8 mm, respectively. Two clearance areas (75 mm × 8 mm), located on the top and bottom sides of the system substrate, were reserved for 4G antennas and other antennas. The relative permittivity of all substrates was 4.4, and the loss tangent was 0.02. Eight antenna elements were evenly placed on two small substrates and fed by eight 50-Ohm connectors through via-holes from the backside of the system substrate. It could also be observed from Figure 1a that four antenna elements were evenly distributed on each small substrate. Antennas 1, 2 (or 5, 6) and 3, 4 (or 7, 8) were symmetrical with respect to the central axis of the system substrate, while antennas 1, 2, 3, 4 and 5, 6, 7, 8 were symmetrical with respect to the central line of the system substrate. Figure 1b shows a side view of the MIMO antenna array. The distance *d_12_* and *d_34_* between the antenna elements were the same, but *d_12_* and *d_23_* were slightly different, which were mainly used to optimize the isolation of the antenna.

### 2.2. Antenna Element Design

**Figure 2:**
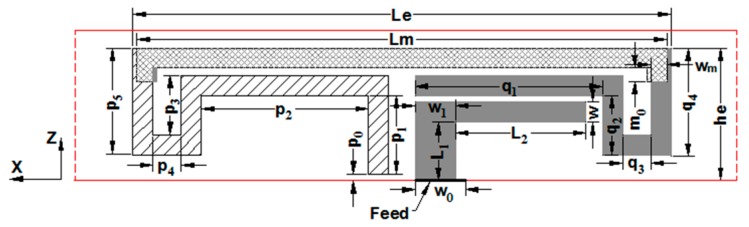
Planar view of the antenna element.

The planar view of an antenna element is shown in Figure 2. Its main structure was a loop antenna printed on both sides of a small dielectric substrate. The gray metal strip was located on the front of the small substrate, and the oblique-shaded metal strip was arranged on the back of the small substrate. The grid-shaped metal strip was the overlapping part of the front and back metal strips, and it could be regarded as a double-sided parallel strip line. By adjusting the width and length of the double-sided parallel strip line, the electromagnetic coupling strength of the front and back metal strips could be changed, thereby regulating the coupling strength of the antenna. This structure was also conducive to miniaturization of the MIMO antenna array. One end of the oblique-shaded metal strip was adjacent to the ground of the system substrate, forming an approximate loop antenna. The analysis showed that, compared with the case of directly contacting with the ground, the adjacent grounding technology could generate an additional resonant frequency to increase the impedance bandwidth of the antenna element. Each loop antenna element was fed by the electromagnetic coupling between the metal microstrip line and the loop, which was beneficial to improving the impedance matching performance of the MIMO antenna.

**Figure 3:**
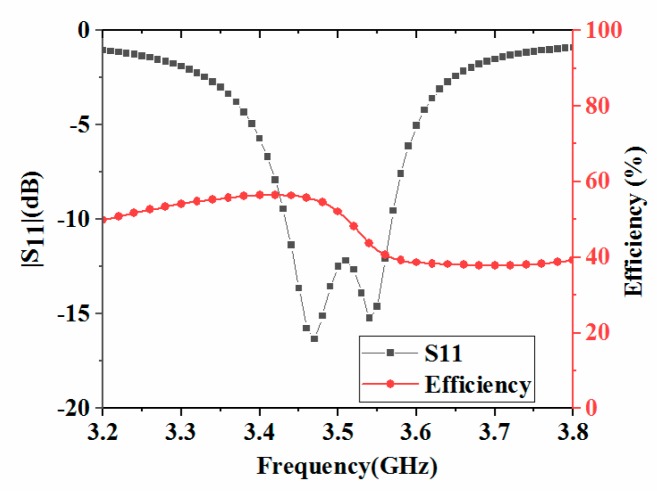
Simulated |S_11_| and radiation efficiency of the antenna element.

To analyze the properties of the antenna element, a loop antenna element was constructed. The system substrate and two small substrates in Figure 1 were retained, and the antenna element structure displayed in Figure 2 was placed on the center axis position of the small substrate. The high frequency structure simulator (HFSS) was used to simulate and optimize the antenna element and MIMO antenna array. Their structural parameters were given as follows: *d_12_* = *d_34_* = 21.9 mm, *d_23_* = 21.9 mm, *f_12_* = *f_34_* = 38 mm, *f_23_* = 40 mm, *L_s_* = 134 mm, *h_s_* = 4.5 mm, *L_e_* = 16.1 mm, *h_e_* = 4 mm, *L_m_* = 15.8 mm, *w_m_* = 0.5 mm, *w_0_* = 1.5 mm, *w_1_* = 1.2 mm, *w* = 0.6 mm, *p_0_* = 0.2 mm, *p_1_* = 2.4 mm, *p_2_* = 5 mm, *p_3_* = 1.8 mm, *p_4_* = 0.8 mm, *p_5_* = 3.2 mm, *q_1_* = 5.6 mm, *q_2_* = 1.8 mm, *q_3_* = 0.8 mm, *q_4_* = 3.2 mm, *L_1_* = 1.8 mm, *L_2_* = 3.9 mm. Figure 3 shows the simulated S-parameters and radiation efficiency of the antenna unit. As could be seen from Figure 3, the antenna had two distinct in-band resonant frequencies, and its −6 dB bandwidth was 3.40–3.59 GHz. The radiation efficiency of the antenna element varied from 38.6% to 56.4% in 3.5 GHz frequency band.

**Figure 4:**
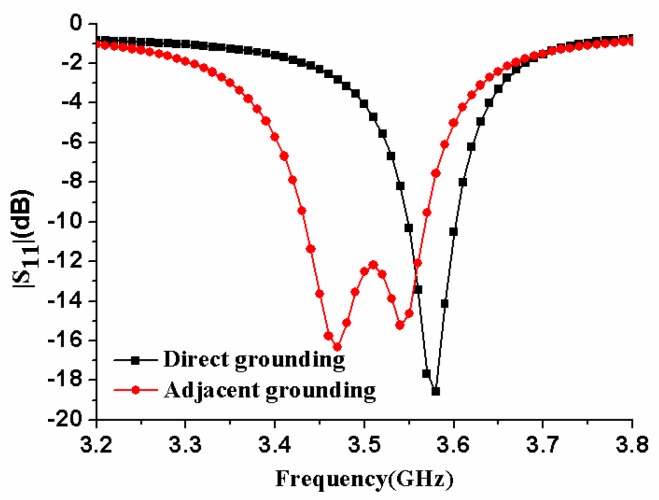
|S_11_| of the antenna element with different grounding methods.

In order to analyze the influences of the relevant structural parameters on the impedance bandwidth of the antenna element, a parametric study had been performed. Figure 4 compares the reflection coefficients of the antenna element in two cases: direct grounding and adjacent grounding. It could be found that there was only one resonant frequency for direct grounding, and two resonant frequencies would be generated for adjacent grounding. Therefore, in order to increase the bandwidth of the antenna element, the way of adjacent grounding was adopted.

**Figure 5:**
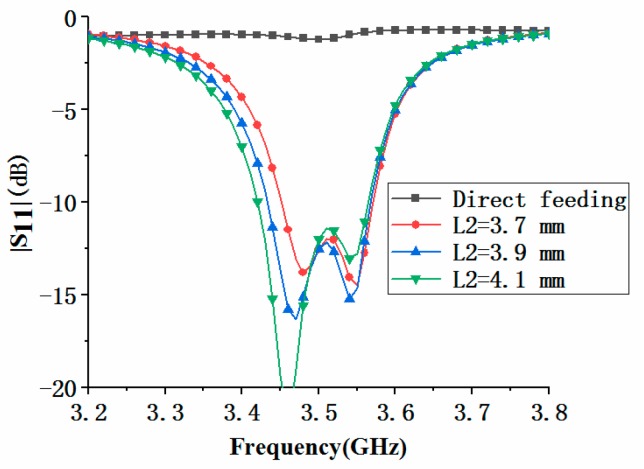
|S_11_| of the antenna element with different feeding methods.

Figure 5 compares the effects of electromagnetic coupling feeding and direct feeding on antenna element performance. Here, the direct feed meant that the microstrip line with a width *w_1_* directly contacted the loop antenna, and the length *L_2_* of the coupling feed line was zero. It could be observed that when the loop antenna was directly fed, impedance matching performance of the antenna element would become very poor. Compared with the direct feeding, the electromagnetic coupling feeding could greatly improve the impedance matching performance of the antenna element. Furthermore, we analyzed the influence of coupling feed line length *L_2_* on antenna performance. When *L_2_* increased, the first resonant frequency moved down, while the second resonant frequency was almost unchanged, so the bandwidth of the antenna became wider.

**Figure 6:**
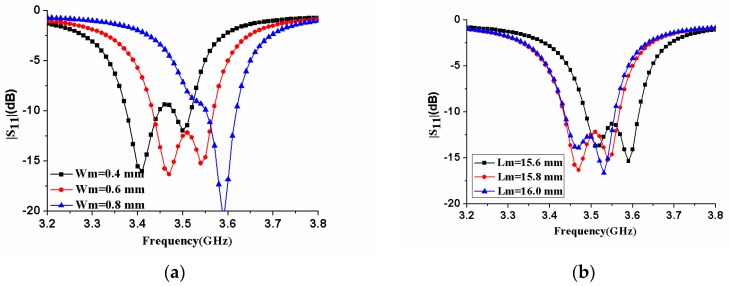
|S_11_| of the antenna element with different sizes of DSPSL (**a**) different width *w_m_*; (**b**) different length *L_m_*.

Figure 6 demonstrates the effect of the double-sided parallel strip line (DSPL) on the reflection coefficients and of the loop antenna element. The analysis showed that the width *w_m_* of the double-sided parallel strip line was directly related to the electromagnetic coupling strength inside the loop antenna. It could be seen from Figure 6a that the larger *w_m_*, the stronger the electromagnetic coupling strength, and the center frequency of the antenna shifted upward. In addition, the length *L_m_* of the double-sided parallel strip line was related to the total size of the loop antenna. As the length *L_m_* became longer, the center frequency of the loop antenna would decrease, as shown in Figure 6b.

**Figure 7:**
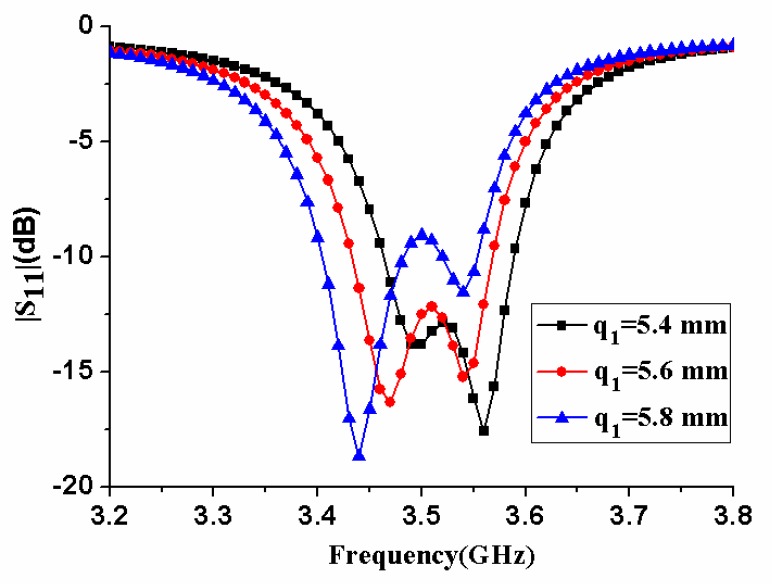
|S_11_| of the antenna element with different size *q_1_*.

**Figure 8:**
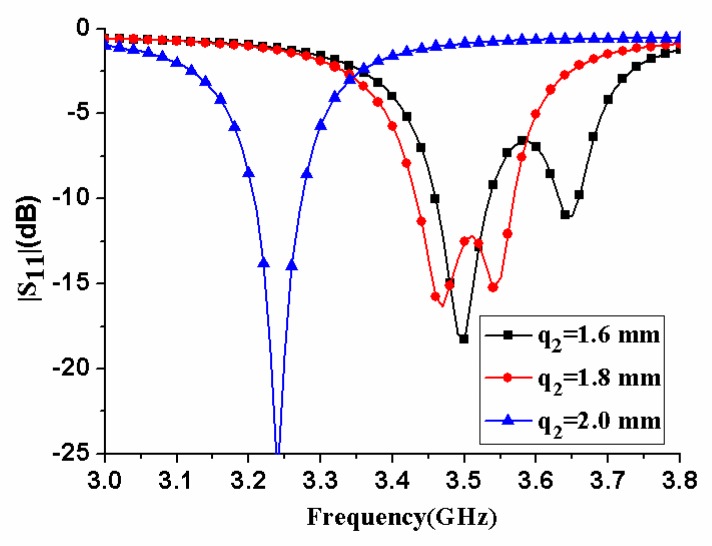
|S_11_| of the antenna element with different size *q_2_*.

Figure 7 displays the effect of antenna element size *q_1_* on antenna performance. When *q_1_* increased, the center frequency of the antenna decreased, and the impedance matching performance deteriorated. In Figure 8, the influence of the antenna element size *q_2_* was analyzed. As *q_2_* increased, the center frequency of the antenna decreased significantly. The antenna’s impedance matching performance improved first, reached an optimal value, and then worsened until one of the two resonance frequencies completely disappeared. Therefore, *q_2_* needed to be selected to a proper value to ensure that the antenna had good impedance matching performance.

## 3. Results and Discussion of the MIMO Antenna Array

**Figure 9:**
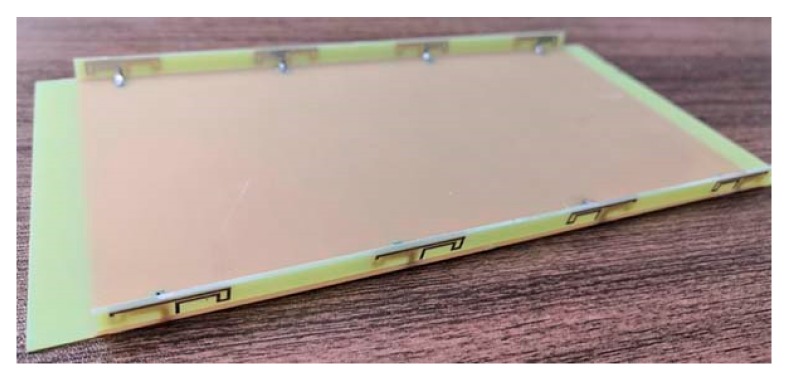
Photograph of the fabricated MIMO antenna array.

Based on the antenna element in Figure 2, an eight-element MIMO antenna array in Figure 1 was fabricated, and its photograph is shown in Figure 9. The S-parameters, radiation performance, and MIMO performance of the MIMO antenna array were given. 

### 3.1. S-parameters

**Figure 10:**
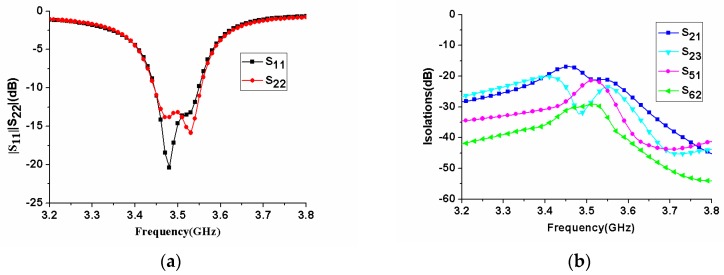
Simulated S-parameters of the proposed MIMO antenna array. (**a**) reflection coefficients; (**b**) isolations.

As the antenna elements of the MIMO antenna array were the same, based on the symmetry of the structure, we only analyzed the reflection coefficients of antenna 1 and antenna 2 and some isolation curves. As shown in Figure 10a, although the positions of antenna 1 and antenna 2 were different, their reflection coefficients remained basically the same. Two distinct resonant frequencies could be observed for each antenna. The common −6 dB bandwidth of antenna 1 and antenna 2 was 3.42–3.58 GHz.

**Figure 11:**
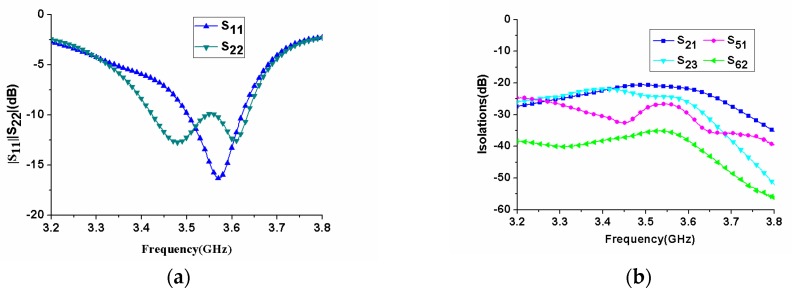
Measured S-parameters of the fabricated MIMO antenna array. (**a**) reflection coefficients; (**b**) isolations.

Based on the arrangement of antenna elements, the isolations between ports 1 and 2, ports 2 and 3, ports 1 and 5, and ports 2 and 6 were given, as shown in Figure 10b. In the figure, it was observed that |S_21_| represented the worst isolation of all isolation curves in the operating frequency band, but it was still better than −17 dB. The S-parameters of the proposed eight-antenna MIMO array were measured by using the Rohde Schwarz zvb20 vector network analyzer (Rohde & Schwarz, Munich, Germany). It could be seen from Figure 11a that the measured in-band return loss of |S_11_| and |S_22_| became worse, but the −6 dB impedance bandwidth became wider, which was 3400–3660MHz, which was wider than the simulated bandwidth. In addition, the maximum measured isolation was better than −20 dB, as shown in Figure 11b. The measured results showed that the proposed MIMO antenna had good isolation between the elements.

**Figure 12:**
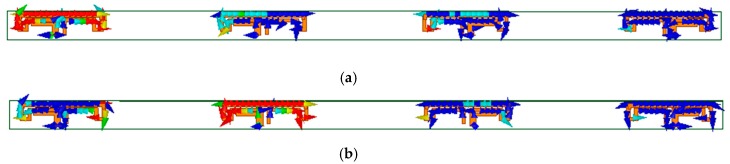
Simulated current distribution at 3.5 GHz of the proposed MIMO antenna array. (**a**) ant 1 excited; (**b**) ant 2 excited.

In order to better understand the high isolation performance of the MIMO antenna, Figure 12 shows the surface current distribution of the MIMO antenna array at 3.5 GHz when antenna 1 and antenna 2 were separately excited. It could be seen from Figure 12 that the current of the antenna was mainly distributed on the exciting element itself and had little effect on other elements, so the antenna elements had high isolation.

### 3.2. Radiation Performances

**Figure 13:**
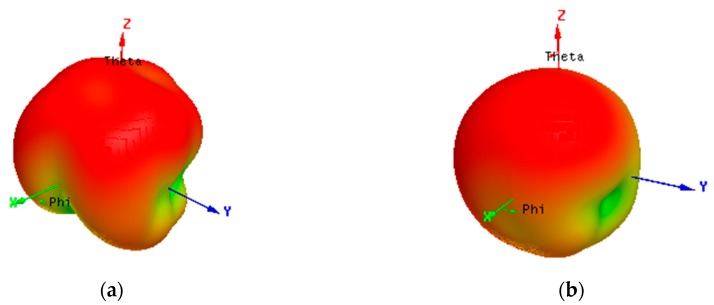
Simulated radiation patterns at 3.5 GHz for the different antenna elements. (**a**) ant 1; (**b**) ant 2.

**Figure 14:**
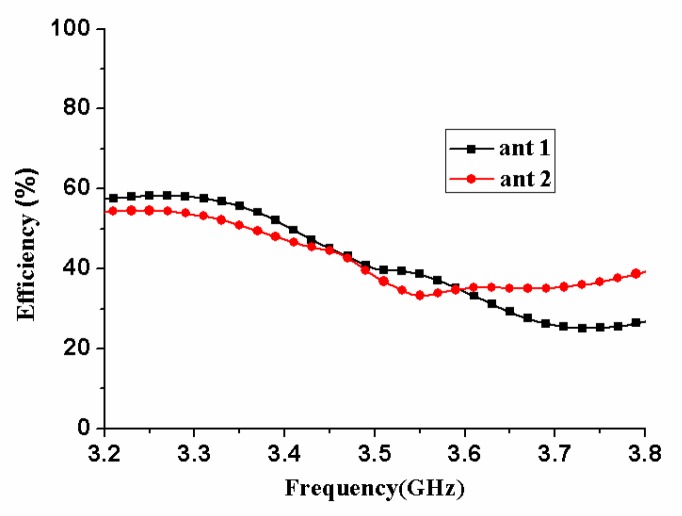
The radiation efficiency of the proposed MIMO antenna array.

Figure 13 shows the radiation pattern of antennas 1 and 2. It could be seen from Figure 10 that their peak gain points were located in different directions, the peak gain point of antenna 1 was near the direction of phi=90°, theta=15°, and the peak gain point of antenna 2 was about phi=0°, theta=30° orientation. Here, the peak gains of antenna elements 1 and 2 were 0.426 and 1.57 dBi, respectively. Figure 14 shows the radiation efficiency of antennas 1 and 2. Their radiation efficiencies were approximately 34%–51% and 33%–47% in 3.4–3.6 GHz band, respectively.

### 3.3. MIMO Performances

**Figure 15:**
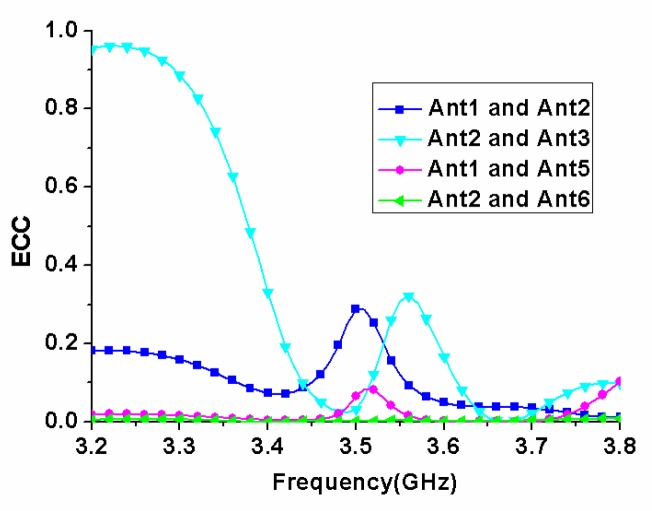
The simulated envelope correlation coefficient (ECC) of the proposed MIMO antenna array.

The envelope correlation coefficient (ECC) is an important parameter to characterize the degree of correlation between channels in a MIMO antenna array, whose theoretical formula is as follows [17]:

(1) $$ECC=\frac{\iint_{4\pi }|({\vec{M}}_{i}(\theta ,\phi ))\times ({\vec{M}}_{j}(\theta ,\phi ))d\Omega {|}^{2}}{\iint_{4\pi }|({\vec{M}}_{i}(\theta ,\phi )){|}^{2}d\Omega \iint_{4\pi }|({\vec{M}}_{j}(\theta ,\phi )){|}^{2}d\Omega }$$

where *M_i_* describes the 3D pattern when the antenna 1 is excited, and *M_j_* describes the 3D pattern when the antenna j is excited. In this work, according to the arrangement of antenna elements in the MIMO antenna array, the ECC curves of antennas 1 and 2, 2 and 3, 1 and 5, 2 and 6 were given, as shown in Figure 15. It could be seen from the figure that in the working frequency band 3.4–3.6 GHz, the simulated maximum ECC value was lower than 0.4, which was smaller than the threshold value 0.5 required for normal operation of the MIMO system. The low ECC value indicated that there was little correlation between antenna elements, and the MIMO antenna array had good MIMO diversity performance.

### 3.4. Parametric Study

**Figure 16:**
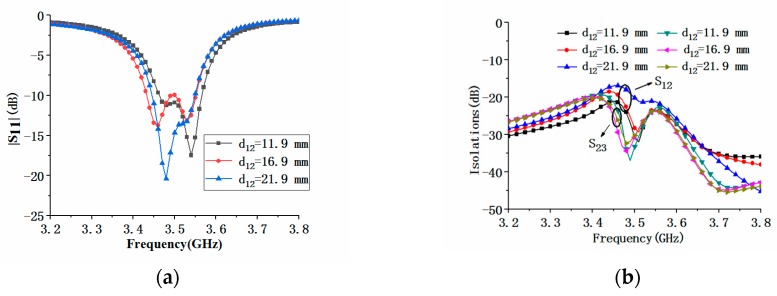
Comparison of |S_11_| and isolation of the proposed antenna with different antenna element spacing *d_12_*: (**a**) |S_11_| comparison; (**b**) |S_21_| and |S_23_| comparison.

**Figure 17:**
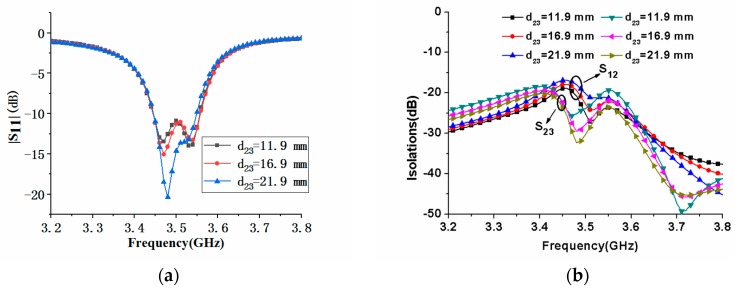
Comparison of |S_11_| and isolation of the proposed antenna with different antenna element spacing *d_23_*: (**a**) |S_11_| comparison; (**b**) |S_21_| and |S_23_| comparison.

In order to better understand the performance of the MIMO antenna array, we analyzed the influence of some representative parameters on its performance. During parameter analysis, only one parameter was changed at a time, and other parameters remained unchanged. Since the proposed MIMO antenna array was composed of eight antenna elements, the position distribution of the antenna elements would affect the performance of the antenna array. Figure 16 shows the effect of the element spacing *d_12_* of the antennas 1 and 2 on |S_11_| and |S_21_|. It could be seen from Figure 16a that when the distance *d_12_* increased, the center frequency of the antenna element was almost unchanged. At the same time, it could be seen from Figure 16b that the changes in the element spacing could affect the isolations between the antenna elements. With the increase of *d_12_*, the isolation between antenna elements 1 and 2 and 2 and 3 decreased, but the degree of reduction was not the same. Comparison of Figure 16 and Figure 17 showed that the influence of antenna spacing *d_23_* on |S_11_| of the MIMO array was similar to that of *d_12_*, but they had different effects on |S_21_| and |S_23_|. As *d_23_* increased, the maximum value of isolation |S_21_| increased, and the maximum value of isolation |S_23_| decreased, as shown in Figure 17. When the antenna element spacing was 21.9 mm, the electric length corresponding to the center frequency of 3.5 GHz was about λ/4.

**Figure 18:**
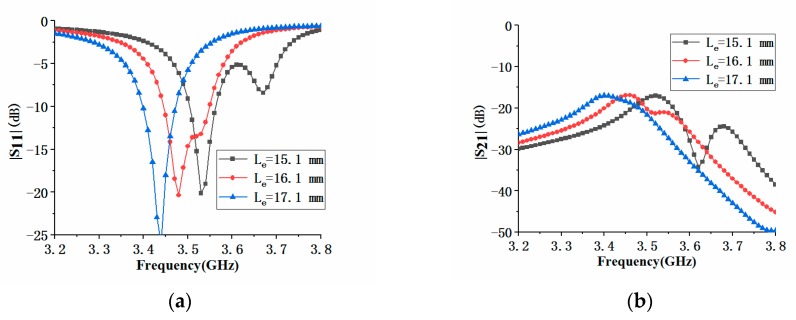
Comparison of |S_11_| and isolation of the proposed antenna with different antenna parameter *L_e_*: (**a**) |S_11_| comparison; (**b**) |S_21_| comparison.

**Figure 19:**
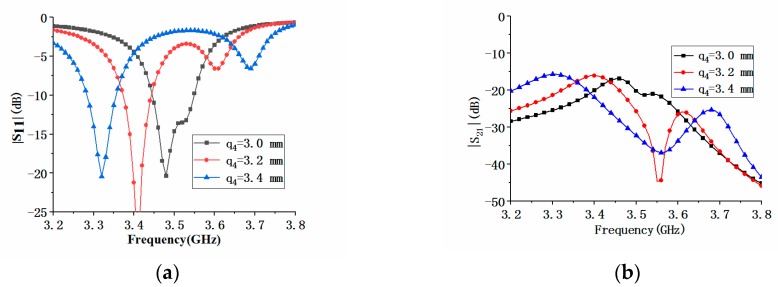
Comparison of |*S*_11_| and isolation of the proposed antenna with different antenna parameter *q_4_*: (**a**) |*S*_11_| comparison; (**b**) |S_21_| comparison.

In addition to the antenna element spacing, we also analyzed the effects of antenna element parameters *L_e_* and *q_4_* on the impedance performance of the MIMO antenna array. Figure 18a shows the effect of the parameter *L_e_* on S_11_ and S_21_. When *L_e_* increased, the center frequency of the antenna element 1 decreased. At the same time, the dip of the first resonance frequency of the antenna became larger, and the dip of the second resonance frequency became smaller until it disappeared. It could be seen from Figure 18b that the increase of *L_e_* had almost no effect on the maximum value of the isolation S_21_, but the maximum value of |S_21_| would move toward the low-frequency direction. Figure 19a shows that the effect of *q_4_* on |S_11_| was similar to that of L_e_, but the effect on |S_21_| was slightly different. When *q_4_* increased, the maximum value of |S_21_| would also move towards the low frequency, but the maximum value would deteriorate, as shown in Figure 19b.

In order to further understand the performance of the proposed antenna, we compared it with other similar 5G frame antennas [27,28,29]. As shown in Table 1, the height of the proposed antenna was 5.3 mm, while the height of other similar frame antennas was 6.8 mm and 7 mm. In addition, the isolations of the proposed antennas were better than −20 dB. Therefore, it could be concluded from Table 1 that the proposed MIMO antenna had a lower-profile and high-isolated performance.

## 4. Conclusions

This paper presented an eight-element MIMO antenna array with a low profile and high isolation. The antenna element was a miniaturized loop antenna. The size of the MIMO antenna array was 150 mm × 75 mm × 5.3 mm. Compared with other similar 5G frame antennas, the proposed antenna had obvious low profile characteristics. Without the use of auxiliary isolation technology, the worst isolation between the antenna elements was better than −20 dB. The proposed MIMO antenna array was constructed and measured. The measured S-parameters were in good agreement with the simulation results and could cover the 3.5 GHz frequency band (3.4–3.6 GHz). In addition, the envelope encapsulation coefficient of the designed MIMO antenna system was less than 0.4, indicating that there was a small correlation between antenna elements. Based on the low profile and high isolation characteristics of the MIMO antenna array, the designed MIMO antenna was suitable for ultra-thin 5G mobile terminal systems.

## Figures and Tables

**Table 1:** Performance comparisons of the multiple-input multiple-output (MIMO) antennas of 5G frame antennas.

Ref.	Size (mm^3^)	−6 dB-BW (MHz)	Isolation (dB)	Efficient (%)
Ref. [27]	150 × 75 × 7.0	3350–3720	−20	60–75
Ref. [28]	150 × 75 × 6.8	3340–3660	−19	50–69
Ref. [29]	150 × 75 × 7.0	3350–3550	−17	45–65
Proposed	150 × 75 × 5.3	3400–3660	−20	33–47
